# Cannabis-opioid interaction in the treatment of fibromyalgia pain: an open-label, proof of concept study with randomization between treatment groups: cannabis, oxycodone or cannabis/oxycodone combination—the SPIRAL study

**DOI:** 10.1186/s13063-023-07078-6

**Published:** 2023-01-27

**Authors:** Cornelis Jan van Dam, Monique van Velzen, Cornelis Kramers, Arnt Schellekens, Erik Olofsen, Marieke Niesters, Albert Dahan

**Affiliations:** 1 Tackling and Preventing The Opioid Epidemic (TAPTOE). consortium, Utrecht, the Netherlands; 2 Department of Anesthesiology, Leiden University Medical Center, Leiden, the Netherlands; 3 Department of Clinical Pharmacy, Canisius-Wilhelmina Hospital, Nijmegen, the Netherlands; 4 Department of Pharmacology‑Toxicology and Internal Medicine, Radboud University Medical Center, Nijmegen, the Netherlands; 5 Department of Psychiatry, Radboud University Medical Center, Nijmegen, the Netherlands; 6 Donders Center for Medical Neuroscience, Donders Institute for Brain, Cognition and Behavior, Nijmegen, the Netherlands; 7 Nijmegen Institute for Scientist-Practitioners in Addiction (NISPA), Nijmegen, the Netherlands; 8 PainLess Foundation, Leiden, the Netherlands

**Keywords:** Chronic pain, Fibromyalgia, Opioids, Cannabis, Adverse effects, Pain relief, Randomized controlled trial

## Abstract

**Background:**

Opioids continue to be widely prescribed for chronic noncancer pain, despite the awareness that opioids provide only short-time pain relief, lead to dose accumulation, have numerous adverse effects, and are difficult to wean. As an alternative, we previously showed advantages of using pharmaceutical-grade cannabis in a population of chronic pain patients with fibromyalgia. It remains unknown whether combining an opioid with pharmaceutical-grade cannabis has advantages, such as fewer side effects from lesser opioid consumption in chronic pain.

**Methods:**

*Trial design*: a single-center, randomized, three-arm, open-label, exploratory trial.

*Trial population*: 60 patients with fibromyalgia according to the 2010 definition of the American College of Rheumatologists.

*Intervention*: Patients will be randomized to receive up to 4 daily 5 mg oral oxycodone sustained release (SR) tablet, up to 5 times 150 mg inhaled cannabis (Bediol®, containing 6.3% Δ^9^-tetrahydrocannabinol and 8% cannabidiol), or the combination of both treatments. Treatment is aimed at self-titration with the daily maximum doses given. Treatment will continue for 6 weeks, after which there is a 6-week follow-up period.

*Main trial endpoint*: The number of side effects observed during the course of treatment using a composite adverse effect score that includes the following 10 symptoms: dizziness (when getting up), sleepiness, insomnia, headache, nausea, vomiting, constipation, drug high, hallucinations, and paranoia.

Secondary and tertiary endpoints include pain relief and number of oxycodone doses and cannabis inhalations.

**Discussion:**

The trial is designed to determine whether self-titration of oxycodone and cannabis will reduce side effects in chronic pain patients with fibromyalgia.

**Trial registration {2a and 2b}:**

EU trial register 2019–001861-33, URL https://www.clinicaltrialsregister.eu, on July 17, 2019; World Health Organization International Clinical Trials Research Platform NL7902, URL https://trialsearch.who.int, on July 26, 2019.

## Administrative information

**Table:** 

**Title {1}**	Cannabis-opioid interaction in the treatment of fibromyalgia pain: an open-label, proof of concept study with randomization between treatment groups: cannabis, oxycodone or cannabis/oxycodone combination – the SPIRAL study
**Trial registration {2a and 2b}**	EU trial register 2019–001861-33, URL https://www.clinicaltrialsregister.eu, on July 17, 2019; World Health Organization International Clinical Trials Research Platform NL7902, URL https://trialsearch.who.int, on July 26, 2019
**Protocol version {3}**	2.0; June 18, 2019
**Funder {4}**	Dutch Research Council (Nederlandse Organisatie voor Wetenschappelijk Onderzoek, NWO) in the framework of the Nationale Wetenschapsagenda—Onderzoek op Routes door Consortia Call (NWA.1160.18.300) and by Bedrocan International BV, Veendam, the Netherlands
**Author details {5a}**	1. Tackling and Preventing the Opioid Epidemic (TAPTOE). consortium, Utrecht, the Netherlands; 2. Department of Anesthesiology, Leiden University Medical Center, Leiden, the Netherlands; 3. Department of Clinical Pharmacy, Canisius-Wilhelmina Hospital, Nijmegen, The Netherlands; 4. Department of Pharmacology‑Toxicology and Internal Medicine, Radboud University Medical Center, Nijmegen, The Netherlands; 5. Department of Psychiatry, Radboud University Medical Center, Nijmegen, The Netherlands 6. Donders Center for Medical Neuroscience, Donders Institute for Brain, Cognition and Behavior, Nijmegen, The Netherlands 7. Nijmegen Institute for Scientist-Practitioners in Addiction (NISPA), Nijmegen, The Netherlands; 8. PainLess Foundation, Leiden, the Netherlands
**Name and contact information for the trial sponsor {5b}**	Leiden University Medical, Center, Leiden, The Netherlands; contact: a.dahan@lumc.nl
**Role of sponsor {5c}**	The principal investigator is the designated trial sponsor; responsible for trial design, execution, analysis and publication of outcomes. The funding parties did not influence trial design, nor will they influence data collection, data analysis, data interpretation or manuscript writing

## Introduction

### Background and rationale {6a}

Opioids, primarily oxycodone, continue to be widely prescribed for chronic noncancer pain (including fibromyalgia), despite the awareness that such medication provides only short-time pain relief, leads to dose accumulation due to tolerance development, has numerous adverse effects, and is difficult to wean due to development of dependence and withdrawal symptoms [1–3]. The TAPTOE consortium, a collaboration of three university centers (Utrecht University, Radboud Medical Center, and Leiden University Medical Center) and SIR, the Institute for Pharmacy Practice and Policy, studies the opioid epidemic in the Netherlands and aims to provide tools to reduce the consumption of opioids for chronic noncancer pain [4]. One possible solution to the opioid problem could be to add a pharmaceutical-grade cannabis variant to the opioid therapy of chronic pain in order to reduce and possibly even eliminate opioid consumption.

We previously showed that inhaled pharmaceutical-grade cannabis, containing both Δ^9^-tetrahydrocannabinol, THC, and cannabidiol, CBD, (the cannabis variety Bediol®), produced a significant reduction of evoked pressure pain in patients with chronic pain from fibromyalgia compared to placebo cannabis [5]. Additionally, the response rate of the reduction in spontaneous pain was significantly greater for the Bediol® cannabis variety containing compared to placebo cannabis or any other cannabis variety (such as varieties containing just THC or just CBD). For this reason, we choose to test Bediol® in the current trial. Bediol® contains 6.3% THC or 63 mg per gram and 8% CBD or 80 mg per gram. Previous studies suggest that using various varieties of cannabis (all non-pharmaceutical grade) as an adjuvant to opioids may reduce pain and opioid consumption in some, but certainly not all patients [6–9]. Additionally, a recent systematic review (2020), involving more than 7000 noncancer patients with chronic pain, found that while opioid dosage decreased in the majority of patients when medicinal cannabis was combined with opioids, all included studies had a high risk of bias [10]. Furthermore, the evidence was insufficient to determine whether cannabis combined with opioids indeed has a favorable outcome in terms of health consequences, and no information could be retrieved on the actual cannabis dose required to reduce opioid consumption [10]. In order to determine whether combining oxycodone with Bediol in chronic pain has advantages over each treatment alone, we will conduct a randomized trial in which patients with fibromyalgia will be randomly assigned to receive either oral oxycodone, inhaled cannabis, or the combination of the two treatments. We chose fibromyalgia patients as this population is commonly treated with potent analgesics including opioids. The study allowed the fibromyalgia patient to self-titrate medication to effect (pain relief and side effects) with a ceiling in amount of drug that may be consumed, and we collected the following data: incidence of side effects (main endpoint), perceived pain levels (secondary endpoint), and opioid and cannabis dose (tertiary endpoint) during the 6-week treatment course and 6-week follow-up period.

### Objectives {7}

#### Primary endpoint

To determine whether self-titration with 150 mg Bediol® (6.3% THC and 8% CBD, inhaled up to 5 times per day) adjunct to self-titration with 5 mg oral oxycodone (up to 2–4 times per day) reduces adverse effects (AEs) as defined by a composite AE score, compared to self-titration with either treatment alone in patients with chronic pain from fibromyalgia.

#### Secondary endpoint

To determine whether self-titration with 150 mg Bediol® (6.3% THC and 8% CBD), inhaled up to 5 times per day adjunct to 5 mg oral oxycodone (up to 4 times per day) reduces pain levels comparable to either treatment alone in patients with chronic pain from fibromyalgia.

#### Exploratory endpoint

To determine whether self-titration with 150 mg Bediol® (6.3% THC and 8% CBD), inhaled up to 5 times per day adjunct to 5 mg oral oxycodone (2–4 times per day) will reduce drug consumption comparable to either treatment alone in patients with chronic pain from fibromyalgia.

## Trial design {8}

The SPIRAL study is an open-label randomized controlled trial. Patients with chronic pain from fibromyalgia will be randomized 1:1:1 to receive daily oral oxycodone, inhaled Bediol®, or oral oxycodone combined with inhaled Bediol®. The study is coordinated by a single center from which the patients receive their medication; all patients will be treated at home. We hypothesize that adverse effects will be less in the groups that receives oxycodone combined with Bediol® (superiority design).

## Methods: participants, interventions, and outcomes

### Study setting {9}

The study will be conducted in the Netherlands, with Leiden University medical Center as coordinating center.

### Eligibility criteria {10}

In order to be eligible for trial participation, a patient must meet all of the following inclusion criteria:

- Chronic pain patients with a pain score ≥ 5 (on a scale from 0 = no pain to 10 = most severe pain imaginable) for most of the day and meet the 2010 American College of Rheumatology diagnostic criteria for fibromyalgia [11]. These criteria include:
  (i) A widespread pain index (WPI) ≥ 7 (on a scale from 0 to 19);
  (ii) And a symptom severity (SymS) score ≥ 5 (on a scale from 0 to 12) or a WPI of 3–6 and a SymS score ≥ 9.

The WPI defines the number of body areas in which a patient experienced pain during the last week; the SymS score indicates the level of other core symptoms of fibromyalgia such as fatigue, non-refreshing sleep and cognitive symptoms.

The following exclusion criteria are being applied:

- Unable to give written informed consent.
- Presence of medical disease that may alter the pharmacokinetics of inhaled cannabinoids or oral oxycodone such as pulmonary or liver disease.
- Allergy to study medication.
- Prolonged (> 3 months) use of strong opioids (oxycodone, fentanyl, buprenorphine, morphine) or tramadol (> 150 mg/day).
- Inability to taper down on previous pain medication in the 4–6 weeks prior to dosing.
- History of illicit drug abuse or alcohol abuse.
- A (family) history of psychosis.
- Pregnancy and/or lactation.
- The presence of pain syndromes other than fibromyalgia.
- Age < 18.

The presence of autonomic complaints such as diarrhea or obstipation, dizziness, and dry mouth/eyes are no reason for exclusion in the chronic pain patient group, as these are symptoms consistent with the fibromyalgia syndrome [12].

### Who will take informed consent? {26a}

After having contacted the coordinating center, all patients that indicate interest in the trial will first receive general information on the trial by the study physician (CJvD). If the patients continue to be interested in participating, they receive a copy of the patient information sheet and consent form. If the patient is willing to participate, oral and written consent will be obtained in person by one of the study physicians (CJvD, AD, MN) before initial screening.

### Additional consent provisions for collection and use of participant data and biological specimens {26b}

Not applicable as no biological specimens will be collected.

## Interventions

### Explanation for choice of comparators {6b}

We will compare adverse effects among the three treatment groups: oxycodone, Bediol®, and the combination of Bediol® and oxycodone. The main reason is that we expect that combining Bediol® with oxycodone will reduce opioid side effects both during treatment and following treatment, i.e., we expect less withdrawal symptoms. We expect that this may partly be due to the expected reduction in opioid dose during Bediol**®** treatment and partly due to a pharmacodynamic interaction. The choice of comparator is based on the fact that adverse effects are the most important limiting factor in the patient compliance.

### Intervention description {11a}

Patients are treated for 6 weeks and randomized to receive one of three treatments:

- Group Oxycodone will receive oxycodone sustained release (SR) 5 mg tablets. In week 1, the patients may dose themselves to a maximum of 2 times 5 mg oxycodone per 24 h. From week 2 on, the patient may increase the dose to a maximum of 4 doses of 5 mg oxycodone if deemed necessary by the patient to subdue their pain.
- Group Cannabis will receive Bediol® 150 mg by inhalation. In week 1, the patients are allowed up to 3 doses per 24 h, the remaining 5 weeks, they are allowed up to 5 inhalations per 24 h.
- Group Cannabis/Oxycodone will receive oxycodone sustained release (SR) tablet (in week 1, the dose is fixed to a maximum of 2 doses of 5 mg oxycodone per 24 h; from week 2 on the patient may increase the dose to a maximum of 4 doses of 5 mg oxycodone) and inhale Bediol® 150 mg (in week 1, just three inhalation sessions are allowed; in weeks 2–6, up to five inhalation sessions in 24 h are allowed).

The 150 mg cannabis flower of the Bediol® variety is placed in a small capsule (pre-packaged by the local university pharmacy) that is placed in a hand-held vaporizer (Mighty Medic vaporizer; Storz & Bickel GmbH & Co, Tuttlingen, Germany). The vaporizer heats the raw material to decarboxylate THC acid and CBD acid into their active forms (THC and CBD, respectively), vaporize the flower, and subsequently cool the vapor so that the cannabis is safely inhaled.

### Criteria for discontinuing or modifying allocated interventions {11b}

The patients are allowed to withdraw their consent at any time during the trial. They will then return their medication to the coordinating center. We anticipate that this may be due to either insufficient pain relief despite the maximal allowed dose or the occurrence of side effects. We will not allow modification of the allocated intervention or increase of the dose above the maximal indicated dosages. In such cases, the patient will be taken out of the study and the medication is returned to the coordinating center. Irrespective of duration of treatment, the data from patients that dropped out after initiation of treatment will be included in the intention-to-treat analysis. Additionally, we will describe into detail the characteristics of the patients that dropped out and reason for dropping out.

### Strategies to improve adherence to interventions {11c}

The patient is contacted weekly by telephone and visits the clinic bi-weekly. Issues related to the course of the study (adherence to medication, side effects, other issues) are discussed and possibly resolved. The patient may contact the investigator at any time by e-mail or telephone to discuss additional issues related to the study.

Medication is provided for no longer than for 2 weeks when they visit the coordinating center. On the third to fifth visit, medication is provided to the patients after they returned their previous stash of medication (oxycodone tablets and/or cannabis). The returned medication will be destroyed.

Patients who drop-out of the study prior or after randomization or prior or after treatment initiation will be replaced by another patient such that we will have three groups of 20 patients that will successfully complete the study. As indicated above, all patients that started on treatment will be included in the intention-to-treat analyses.

### Relevant concomitant care permitted or prohibited during the trial {11d}

The patient may not consume any other opioid medication than those provided by the coordinating center during the course of the 12-week study period. Paracetamol and medication for treatment of neuropathic pain (including gabapentinoids) are allowed when used for more than 6 weeks at a steady dose. Concomitant medication will be recorded. Also non-pharmacological interventions are allowed.

### Provisions for post-trial care {30}

The general practitioner will be involved in post-trial care such that the patient will remain on adequate pain treatment. Additional issues will be resolved by the research team and general practitioner.

### Outcomes {12}

#### Main trial endpoint

The main study outcome is the number of side effects observed during the course of treatment. To that end, we constructed a composite adverse effects (cAE) score. The cAE score includes the following 10 symptoms: dizziness (when getting up), sleepiness, insomnia, headache, nausea, vomiting, constipation, drug high, hallucinations, and paranoia. The subjects will score all of these symptoms at the end of each day of treatment on paper or within the electronic data capture system Castor (Castor EDC, Ciwit BV, www.castoredc.com). Each symptom will result in 1 point (min. score per day = 0, i.e., there were no symptoms; max. score per day = 10, i.e., all symptoms occurred) for the 42 days of treatment. The maximum score for the complete 42-day treatment period is 420.

#### Secondary endpoint

The secondary endpoint of the study is pain perception. We scored spontaneous pain (i.e., the pain related to fibromyalgia) on a daily basis and the response to experimentally induced pain on a bi-weekly basis. The pain diary will be collected through the web-based data entry system, or on paper forms.

##### Spontaneous pain score

There are five questions asked that the patient will answer at the end of the day (around diner time):

- Question 1: How much pain did you experience on average over the last 24 h (score 0–10);
- Question 2: What was the maximum pain that you experienced over the last 24 h (score 0–10);
- Question 3: How satisfied are you with the treatment over the last 24 h (score 0–10);
- Question 4: How dissatisfied are you with your pain over the last 24 h (score 0–10);
- Question 5: How do you rate the treatment in relation to the side effects (score 0–10);

For questions 1 and 2: score 0 = no pain, score 10 = the most severe pain imaginable;

For questions 3 to 5: score 0 = not satisfied at all, score 10 = very satisfied with this treatment.

At the end of the treatment period, we will ask these same questions but then for the complete treatment period. During follow-up, we will continue asking questions 1 and 2 on a weekly basis for 6 weeks. Additionally, we will ask the patient about pain medication use in the follow-up period.

##### Quantitative sensory testing

Bi-weekly (during their visit to the coordinating center), we will apply two experimental tests to determine the pain system of the patient. Pain test 1 is a pressure pain threshold test. Pain test 2 tests pain summation to repetitive pinprick stimuli (wind-up). For pain pressure, we will use the FDN 100 N Algometer (FDN 100, Wagner Instruments Inc., Greenwich, CT, USA). The algometer is used to deliver pressure pain on a skin area of 1 cm^2^ between thumb and index finger. The FDN 100 has a force capacity (± accuracy) of 100 ± 2 N (10 ± 0.2 kgf) and graduation of 1 N (100 gf), respectively. A gradually increasing pressure is manually applied, and the subjects are asked to indicate when the procedure becomes painful (pressure pain threshold).

Pain summation is obtained by applying a pin prick stimulator (MRC Systems, Germany) to the dorsal part of the hand. The patient will be requested to score the pin pricks on a 10-point numerical rating scale (NRS) ranging from 0 (no pain) to 10 (most severe pain imaginable). We will collect the NRS of the first and the last poke. An increase in NRS over the course of poking is a sign of central sensitization.

#### Exploratory outcome

The number of medication administrations will be collected on a daily basis. For oxycodone, in week 1, a maximum number of tablets consumed per day is 2, and for weeks 2–6, 4 tablets may be consumed per day. This makes a total of 154 tablets for the 6-week treatment period. For Bediol®, the maximum number of inhalations is 3 per day in week 1, and 5 in weeks 2–6. In total, 196 inhalation episodes are possible. These data will be collected daily through the web-based data entry system, or on paper forms.

#### Other outcomes

At baseline, we will perform a cornea confocal examination to quantify the C-fibers of the cornea [13], in order to get an indication of the presence of small fiber neuropathy in our patient population. This will allow us to associate drug effects with patient phenotypes based on the cornea C-fiber state. At baseline, we will also perform the two experimental pain tests (see above).

Patients will be queried as follows prior, during or after the treatment period:

- At screening: the MINI plus, a neuropsychiatric questionnaire aimed to detect active psychiatric problems. This questionnaire will be taken at screening. In case of issues the patient will be referred to his/her family doctor.
- During the bi-weekly visit to the coordinating center during the course of treatment:
  1. A score for opioid and/or cannabis craving (depending on the treatment).
  2. Hospital Anxiety and Depression Scale (HADS) questionnaire.
  3. The Current Opioid Misuse Measure (COMM) questionnaire.

- During follow-up (i.e., after the 6-week treatment) bi-weekly:
  1. The OOS (“objective withdrawal scale”) questionnaire.
  2. The SOS (“subjective withdrawal scale”) questionnaire.
  3. A score for opioid or cannabis craving (depending on the treatment).
  4. Hospital Anxiety and Depression Scale (HADS) questionnaire.
  5. The Current Opioid Misuse Measure (COMM) questionnaire.

- And finally, at screening, end of treatment, and end of study, the MATE-Q, which gives an indication of drug use [14].

### Participant time line {13}

**Fig. 1:**
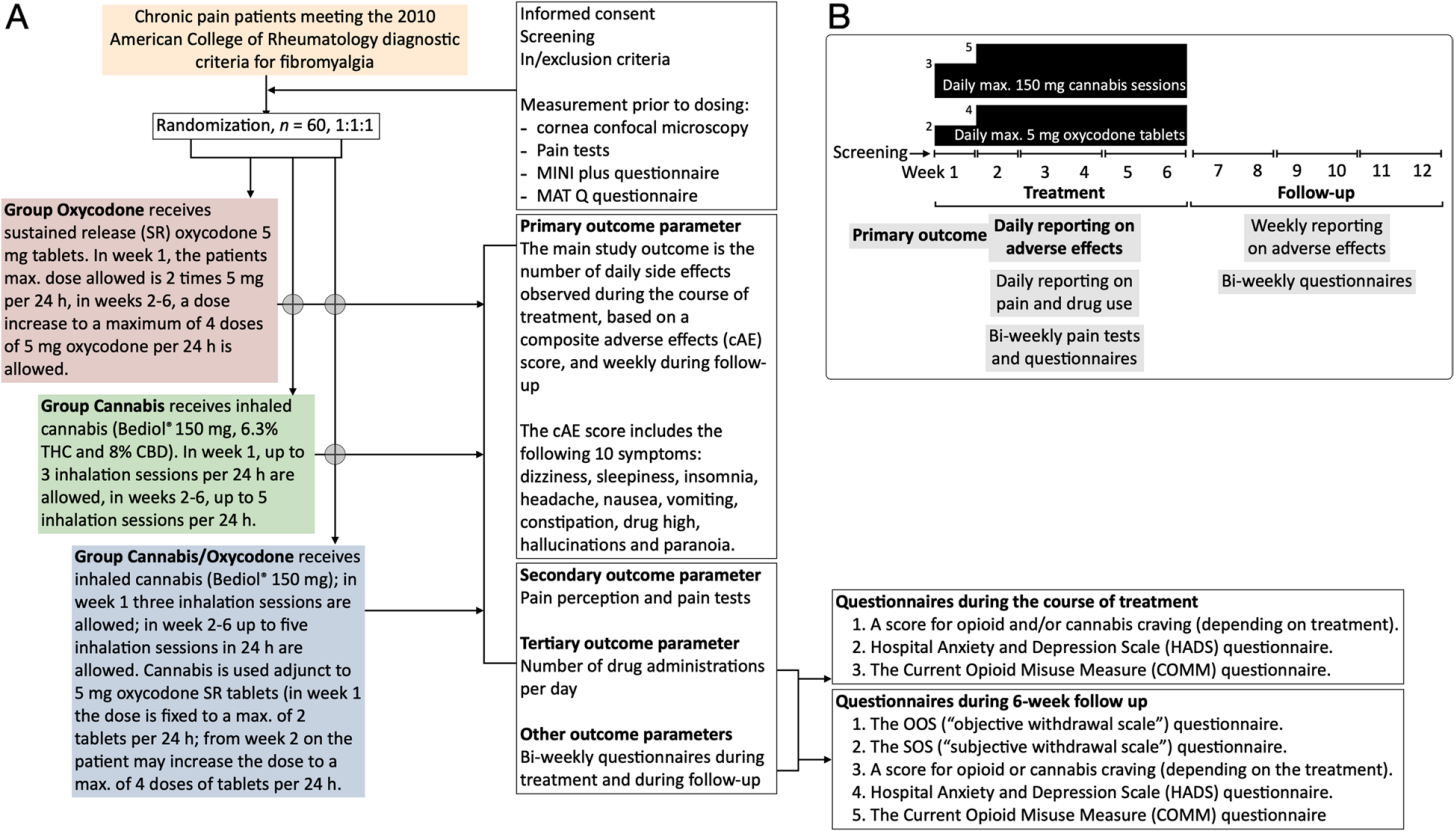
**A** Study procedures flow diagram. **B** Timing of dosing and measurements of endpoints

A schedule of trial enrolment, interventions, and outcomes is given in Fig. 1.

### Sample size {14}

Sample size calculations were based on the primary outcome—the composite adverse effects (cAE) score. A power analysis was performed using Monte Carlo simulation. In brief, the count of side effects data was assumed to be binomially distributed. One hundred simulated data sets were fitted to a mixed effects model (using glmer in R), and the number of times the *p*-values for the test for significance of the interaction term (time*treatment) were below alpha = 0.05 was counted. In the opioid-only group, the baseline counts were assumed to be around 5 and unchanging over time; in the group cannabis/oxycodone, the counts were assumed to decrease by 20% over time to 4. Inter-occasion plus interindividual variability of the time*treatment effect was assumed to have a standard deviation of 0.3 at the end of the 6 weeks [5]. For a power of around 80%, 20 subjects in each arm will be needed. The sample size analysis was conducted in NONMEM, a statistical program for non-linear mixed effects modeling after consultation with the department of statistics of the Leiden University Medical Center (dr. Roula Tzonakis). In the analysis, we restricted our comparison to the opioid *versus* opioid/oxycodon group as we intend to study the effect of adding cannabis to opioid treatment on side effects. The third group, cannabis only, was added to allow exploratory comparisons with the other two groups.

### Recruitment {15}

Patients will be recruited via dedicated forums for chronic pain patients and fibromyalgia, via the pain clinic of the coordinating center, and via recruitment ads in local newspapers. Expected recruitment will be 1–3 patients per month and expected duration of the study is 24 months.

## Assignment of interventions: allocation

### Sequence generation {16a}

Patient will be randomized (1:1:1, without stratification or block design) to treatment on the second visit to the coordinating center, when they receive their first set of medication. The randomization sequence is generated by a dedicated computer randomization software, Castor (Castor EDC, Ciwit BV, www.castoredc.com).

### Concealment mechanism {16b}

This is an open-label trial, with randomization to treatment performed using a computer-generated randomization list. Use of a validated password website using Castor will ensure concealment.

### Implementation {16c}

The generation of the allocation sequence, randomization, and assignment of participants to treatment is done by one of the study coordinators/supervisors.

## Assignment of interventions: blinding

### Who will be blinded? {17a}

This is an open-label non-blinded study.

### Procedures for unblinding if needed {17b}

The design of the study is open label with only outcome assessors being blinded so unblinding will not occur.

## Data collection and management

### Plans for assessment and collection of outcomes {18a}

All data are collected in the electronic data capture system CASTOR (Castor EDC, CIWIT B.V., www.castoredc.com). After obtaining informed consent, we will collect the following variables at baseline: age, sex, height (cm) and weight (kg), blood pressure (mmHg) and heart rate (beats/min), fibromyalgia related variables (WPI and SymS), duration of disease, current pain score, wind-up, and pain threshold. Subsequently at various intervals, we will collect the following: daily adverse events on a 10-point score, daily pain score, daily drug use, and bi-weekly wind-up and pain threshold, and information retrieved from the questionnaires (see section Outcomes {12}) prior and during treatment and during follow-up at 2-week intervals.

### Plans to promote participant retention and complete follow-up {18b}

Patients that fail to input their data in the electronic data capture system will be actively approached and aided in the completing of data entry.

### Data management {19}

A pseudonymization key file containing patient identities will be stored on a secure partition of the computer system of the coordinating center after approval of the data protection officer of the coordinating center; data confidentiality will be protected by measures outlines in paragraph {27}. Outcome data is collected in the electronic data capture system CASTOR (www.castoredc.com), which is intentionally designed to record trial data and has an audit log trail. The system allows data entry by both the researcher and patient through a web-based interface. To reduce data input errors, data will be range-checked during data entry; if data falls outside the intended range, a stop error will occur. Source data will be retained at the coordinating center. Only authorized investigators (CJvD, MvV, MN, AD) are permitted to amend or complete data input, with all corrections will be logged in an audit log trail. All data will be handled confidentially and in a pseudonymized fashion. The database is closed after it has been certified complete and accurate. Data processing is the responsibility of the sponsor, researchers, and project leader. If a subject withdraws consent, data acquired up to that point may be used. All data will be retained for the duration of the trial and for 25 years afterwards for further analyses and publication. The Dutch Personal Data Protection Act will govern all personal data management.

### Confidentiality {27}

All patients will have a random subject identification code that does not include any patient identifying data. The encrypted codebook will be digitally kept and will be safeguarded by the study physician. Other involved parties (data monitoring committee, Health and Youth Care Inspectorate) may be permitted access to patient data (including patient identifiers). This may be necessary to determine patient safety or whether the study is conducted in a safe manner. These involved stakeholders will maintain the confidentiality of identifying patient data.

### Plans for collection, laboratory evaluation, and storage of biological specimens for genetic or molecular analysis in this trial/future use {33}

There will be no biological specimens collected.

## Statistical methods

### Statistical methods for primary and secondary outcomes {20a}

A mixed effects model will be used to analyze the data, with number of adverse events or pain score as dependent variable, and time, treatment, and treatment × time as fixed effects, and post hoc comparison between Groups Oxycodone and Oxycodone/Cannabis as main comparison; *p*-values < 0.05 are considered significant. We will analyze the different components of the composite AU score. See protocol for details (available at a.dahan@lumc.nl).

### Interim analyses {21b}

There are no interim analyses planned. Termination of the study depends on the ruling of trial steering committee and monitor and such a decision will be made in close collaboration with the medical research and ethics committee. It will be based primarily on the occurrence of serious or unsuspected adverse events (see below).

### Methods for any additional analyses {20b}

The exploratory end point, drug use, will be analyzed by comparing total number of oxycodone tablets used between oxycodone and oxycodone/cannabis and total number of cannabis inhalations between the cannabis and oxycodone/cannabis groups. These later comparisons are considered exploratory and no alpha-value will be a priori set for level of significance. All other outcome variables will not be compared but presented as either mean ± SD, median (interquartile range), number, or percentage, as deemed appropriate. Additionally, we will analyze the different components of the composite AU score. For example, we will separate opioid from cannabis side effects (e.g., hallucinations, paranoia) and analyze these separately.

### Methods in analysis to handle protocol non-adherence and any statistical methods to handle missing data {20c}

The data from all enrolled patient (*n* = 60) that do not drop out of the study prior to reaching end-of-follow-up will be analyzed. Missing measurements will not be inputted in the data analysis. Analysis of data from patients that do not complete the study will be performed as well, as we believe that these data are valuable and possibly give an indication for the reasons of dropping out.

### Plans to give access to the full protocol, participant-level data, and statistical code {31c}

The protocol, participant-level data sets, and statistical codes are available from the corresponding author after agreement is reached regarding purpose of such request and proposed analyses and publication strategy.

## Oversight and monitoring

### Composition of the coordinating center and trial steering committee {5d}

The coordinating center and trial steering committee is composed of 3 study physicians and one study coordinator that will oversee patient recruitment and enrolment, the progression of recruitment, evaluation of adverse events, and monitoring of subject drop-out. The committee will convene one every 2 weeks during the study during which one participant of the committee will give a presentation with all current data regarding the trial. After completion of the study, the committee will become the data management team and assess completion of data collection and will prepare the data files for analyses.

### Composition of data monitoring committee, its role and reporting structure {21a}

The study will regularly be monitored by a trained and independent monitor. Monitoring will consist of the control of the presence and completeness of all research files and informed consent forms. According to the monitoring plan, source data checks will be conducted at regular intervals. The monitor will report directly to the principal investigator. Additional monitoring details are specified in a dedicated monitoring plan.

### Adverse events reporting and harms {22}

Adverse events (AEs) are defined as any undesirable experience occurring to a subject during the trial, whether considered related to the trial procedure. A serious adverse event (SAE) is any untoward medical occurrence or effect:

- Results in death.
- Is life threatening (at the time of the event).
- Requires hospitalization or prolongation of existing inpatients’ hospitalization.
- Results in persistent or significant disability or incapacity.
- A congenital anomaly or birth defect.
- Any other important medical event that did not result in any of the outcomes listed above due to medical or surgical intervention but could have been based upon appropriate judgment by the investigator. An elective hospital admission will not be considered as a serious adverse event.

All (S)AEs reported spontaneously by the subject or observed by the investigator or his staff will be recorded, only if judged to be substantial deviating from expected standard clinical course. The investigator will report all SAEs to the sponsor without undue delay after obtaining knowledge of the events. Subjects will be followed up for AEs and SAEs until the final trial procedures or 7 days after discontinuation of the trial. All reports will be digitally filed in the electronic clinical data capture form.

The sponsor will report the SAEs to the accredited Medical Research and Ethics Committee (MREC) Leiden-Den-Haag-Delft, within 7 days of first knowledge for SAEs that result in death or are life threatening followed by a period of maximum of 8 days to complete the initial preliminary report. All other SAEs will be reported within a period of maximum 15 days after the sponsor has first knowledge of the serious adverse events.

### Frequency and plans for auditing trial conduct {23}

Random audits of trials performed by the sponsor are allowed by an independent auditing committee. No auditing is scheduled in advance.

### Plans for communicating important protocol amendments to relevant parties (e.g., trial participants, ethical committees) {25}

A protocol amendment is defined as an amendment to the terms of the MREC application or to the protocol or any other supporting documentation that is likely to affect to a significant degree:

- The safety or physical or mental integrity of the subjects of the trial.
- The scientific value of the trial.
- The conduct or management of the trial.
- The quality or safety of any intervention used in the trial.

Any protocol amendment will not be implemented prior to MREC approval and will be communicated with the trial participants and the trial registries.

### Dissemination plans {31a}

The protocol is available from the authors (a.dahan@lumc.nl). In accordance with the Central Committee on Research Involving Human Subjects (CCMO) statement on publication policy, the results of this trial will be disclosed unreservedly, including to trial participants.

## Discussion

Given their great efficacy, particularly in the early weeks to months of treatment, opioids remain an important tool in the management of chronic pain. For example, in the Netherlands alone, more than one million inhabitants are prescribed an opioid for treatment of pain [1–3]. The adverse effects that arise during opioid treatment are a matter of great concern. Side effects of physician-supervised opioid consumption include dizziness/lightheadedness, sedation, insomnia, headache, nausea, vomiting, constipation, and drug high. We designed a study that attempts to curb the side effect profile observed during opioid treatment. We do so by combining oxycodone treatment with the inhalation of pharmaceutical-grade cannabis, assuming that the addition of cannabis will reduce the opioid dose and consequently the number of side effects decreases. We are aware that cannabis treatment itself can also come with side effects such as drug high, sedation, and possibly hallucinations and paranoia. However, in this trial, the patient will self-titrate the medication to effect and side effects, and we therefore envision that side effects will remain mild to moderate in severity. We expect patients to curb dosing when AE increase in severity. Moreover, we designed a composite score of occurrences of adverse events that includes both opioid and cannabis side effects. While this seems a reasonable approach, this directly points towards two limitations. First, it may well be that the combination of oxycodone and cannabis leads to a reduction in dose of either component (compared to treatment of either component alone) without changing combined AE profile. Second, we do not score for the severity of each AE component. This is done to simplify data analysis. Additionally, we are aware that patients are able to determine whether an AE is present (such as dizziness) but have difficulty in scoring the severity of such symptoms. Hence, a more practical approach was chosen.

Another limitation of the study is that by combining an opioid with a cannabis product, we might increase the likelihood of abuse and addiction. However, the drug use will be strictly monitored, and hence we expect no drug abuse. Additionally, the chosen dosages are relatively low precluding serious adverse events such as respiratory depression or sedation. In fact, in a recent study (EU trial register, https://www.clinicaltrialsregister.eu, identifier 2021–000083-29), we tested the combination of THC and 20 mg oxycodone in opioid-naïve volunteers and observed absence of enhancement of opioid-induced respiratory depression, and in some subjects even some respiratory stimulation from THC, probably related to its effect at the cannabinoid type 2 receptor in brainstem respiratory networks.

The study is not blinded, and absence of blinding might cause bias that influences study outcome. We reasoned that blinding of the trial would complicate the trial in such a way that it would become impractical to perform. A double dummy approach with placebo tablets (as replacements of oxycodone) and placebo cannabis (as replacement of Bediol®) is theoretically possible but in practice expensive and difficult to manage. We therefore chose the current approach and consider this a proof of concept or exploratory protocol that will lay the basis of future protocols.

An important issue is the risk of diversion of medication. To prevent diversion, the patients were instructed to return all unused medication to the researched on their two-weekly visits. Use of opioid medication other than oxycodone during the study was not allowed. We did not test the subjects for compliance with this restriction. For both items, we rely on the trust between the physician researcher and patient that was created by the close contact between the two. Still, a full-proof strategy to eliminate diversion or additional opioid use is not in our setup.

In conclusion, we will study the effect of oxycodone in combination with inhaled cannabis (the Bediol® variety) relative to either treatment alone, on adverse events during a 6-week treatment course. We are aware that this is a rather complex trial with a large set of outcomes apart from the single primary endpoint, the AE composite score. We believe that this is an important trial, however, that will shed light on our ability to curb opioid treatment for chronic noncancer pain.

## Trial status

Trial recruitment started on July 26, 2019, but the trial was delayed because of problems in the cannabis production process and due to COVID-19 restrictions in performing non-COVID-19 medical research in the coordinating center (LUMC). The recruitment is therefore still ongoing. Completion of the study is expected in mid 2023.

## Data Availability

The final data set is available in full to the investigators (AD, CJvD, MN, MvV, EO, CK, and AS). There are no contractual agreements that limit access for the investigators.

## Abbreviations

cAE: Composite adverse events score
CBD: Cannabidiol
CCMO: Central committee on research involving human subjects
COMM: Current opioid misuse measure
HADS: Hospital Anxiety and Depression Scale
LUMC: Leiden university medical center
MREC: Medical research and ethics committee
NONMEM: Non-linear mixed effects modeling
NRS: Numerical rating scale
OOS: Objective withdrawal scale
SIR: Institute for pharmacy practice and policy
SOS: Subjective withdrawal scale
SR: Sustained release
SymS: Symptom severity score
TAPTOE: Tackling and preventing the opioid epidemic
THC: Δ^9^-Tetrahydrocannabinol
TSC: Trial steering committee
WPI: Widespread pain index
